# Le tophus goutteux

**DOI:** 10.11604/pamj.2014.17.250.3223

**Published:** 2014-04-07

**Authors:** Kawtar Inani, Fatimazahra Mernissi

**Affiliations:** 1 Service de Dermatologie, Chu Hassan II, Fès, Maroc

**Keywords:** Tophus, goutte, nodules sous cutanés, tophi, gout, subcutaneous nodules

## Image en medicine

Le tophus correspond à des dépôts sous cutanés d'urate, C'est l'expression ostéo-articulaire d'une hyper uricémie, manifestation actuellement rare. Cliniquement, il se manifeste par des nodules sous cutané, de consistance ferme, volumineux soulevant un épiderme aminci, et qui se localisent sur les petites articulations distales des mains et des pieds, mais aussi au niveau des coudes, des genoux et des cartilages auriculaires. L'ulcération des tophus est rare, ainsi que leur surinfection, puisque l'acide urique constitue un milieu défavorable à la pullulation microbienne. Le diagnostic différentiel se pose essentiellement avec le pilomatricome, les calcinoses tumorales et les calcinoses par anomalies métaboliques phosphocalciques. Nous rapportons l'observation d'un Patient de 70 ans, sans antécédents pathologiques notables, qui consultait pour des lésions nodulaires acrales, évoluant depuis un an avec notion d'ulcération et issue de bouillie crayeuse. L'examen clinique trouvait des nodules au niveau des deux mains de consistance dure, à surface irrégulière, adhérents au plan profond. Le bilan biologique a objectivé une insuffisance rénale, un taux d'acide urique à 150 mg/l, et un bilan phosphocalcique normal. les radiographies standards étaient normales. Le patient fut mis sous régime avec colchicine et allopurinol au long court. L’évolution était marquée par une régression progressive des nodules sous cutanés.

**Figure 1:**
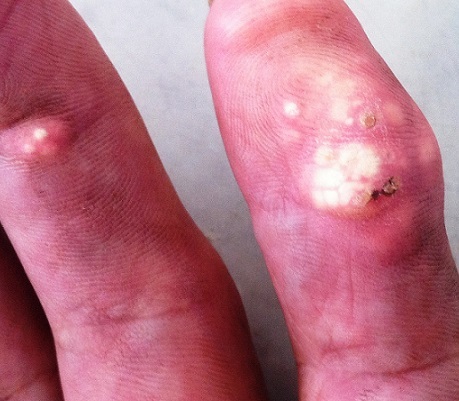
Nodules sous cutanés de consistance dure, en regard des articulations des mains, avec peau en regard aminci

